# *De novo*-generated small palindromes are characteristic of amplicon boundary junction of double minutes

**DOI:** 10.1002/ijc.28084

**Published:** 2013-03-09

**Authors:** Jing Zhu, Yang Yu, Xiangning Meng, Yihui Fan, Yu Zhang, Chunshui Zhou, Zhichao Yue, Yan Jin, Chunyu Zhang, Lisa Yu, Wei Ji, Xueyuan Jia, Rongwei Guan, Jie Wu, Jingcui Yu, Jing Bai, Xin-Yuan Guan, Mingrong Wang, Ki-Young Lee, Wenjing Sun, Songbin Fu

**Affiliations:** 1 Laboratory of Medical Genetics, Harbin Medical UniversityHarbin, People's Republic of China; 2 Key Laboratory of Medical Genetics (Harbin Medical University), Heilongjiang Higher Education InstitutionsHarbin, People's Republic of China; 3 Department of Clinical Oncology, University of Hong KongHong Kong, People's Republic of China; 4 State Key Laboratory of Molecular Oncology, Cancer Institute (Hospital), Chinese Academy of Medical Sciences and Peking Union Medical CollegeBeijing, People's Republic of China; 5 Department of Cell Biology & Anatomy, University of CalgaryCanada

**Keywords:** gene amplification, double minutes, junction sequence, amplicon boundary palindrome, cancer

## Abstract

Double minutes (DMs) are hallmarks of gene amplification. However, their molecular structure and the mechanisms of formation are largely unknown. To elucidate the structure and underlying molecular mechanism of DMs, we obtained and cloned DMs using microdissection; and degenerated oligonucleotide primed polymerase chain reaction (DOP-PCR) from the ovarian cancer cell line UACC-1598. Two large amplicons, the 284 kb AmpMYCN, originating from locus 2p24.3 and the 391 kb AmpEIF5A2, from locus 3q26.2, were found co-amplified on the same DMs. The two amplicons are joined through a complex 7 kb junction DNA sequence. Analysis of the junction has revealed three *de novo* created small palindromes surrounding the six breakpoints. Consistent with these observations, we further found that 70% of the 57 reported DM junction sequences have *de novo* creation of small palindromic sequences surrounding the breakpoints. Together, our findings indicate that *de novo*-generated small palindromic sequences are characteristic of amplicon boundary junctions on DMs. It is possible that the *de novo*-generated small palindromic sequences, which may be generated through non-homologous end joining in concert with a novel DNA repair machinery, play a common role in amplicon rejoining and gene amplification.

Gene amplification is a form of genomic aberration found in many tumors, and it is associated with tumor development and drug resistance.^1^ Gene amplification is cytogenetically manifested as intra-chromosomal homogeneously staining regions (HSRs) or extra-chromosomal double minutes (DMs).^2^ However, the molecular architecture and underlying mechanisms for gene amplification are still poorly understood. In human ovarian cancer cell line UACC-1598, a 3q26 amplicon on DMs carrying a novel candidate oncogene *EIF5A2* was identified.^3^ The more detailed molecular structure of DMs is unknown. Research into the molecular structure of DMs will provide a basis for potential carcinogenetic gene identification and throw light on the molecular mechanisms of gene amplification.

On the chromosomal level, the breakage-fusion-bridge (BFB) has been recognized as a common mechanism for gene amplification.^4^ The BFB cycle is triggered by spontaneous double-strand breaks (DSBs) during chromosome segregation, followed by fusion of broken sister chromatids, which generates a dicentric chromosome.^5^ This dicentric chromosome segregates into different daughter cells and results in new chromosomal breaks during next mitotic segregation, which triggers additional rounds of amplification via BFB cycles. Eventually, the chromosome is stabilized by addition of a telomere.^6^ In addition, the BFB cycle plays an important role in generating chromosomal aberrations including formation of DMs in cancer cells.^7^

However, the BFB cycle does not address the mechanism by which the chromosomal fragments are fused. Studies on boundary palindromes have provided more insights into the molecular process of gene amplification.^8^–^10^ In *Tetrahymena*, a 42 bp inverted repeat adjacent to a break site forms a large palindrome and promotes palindromic amplification of the rDNA by fold-back primed DNA synthesis.^11^ In yeast and mammalian Chinese hamster ovary (CHO) cells, artificially engineered constructs with inverted repeats adjacent to DSBs also confer amplification advantages.^12^,^13^ The drug-resistance related gene *DHFR* is associated with large DNA palindromes, and an initial palindromic duplication of *DHFR* triggers the BFB cycle and subsequent gene amplification.^5^,^13^,^14^ All these experimental observations suggest that formation of large palindromes can be an early event for gene amplification.^13^,^15^

Palindrome formation is dependent on the nature of the inverted repeat DNA sequence in a genomic region. In general, palindromes can be divided into three subtypes, including perfect palindromes, spaced inverted repeats, and imperfect inverted repeats with different secondary architecture and distinct outcomes of genome instability.^9^ True palindromes form stem type only hairpin structures, also cause cruciforms, which lead to genomic instability.^16^ Spaced inverted repeats are more natural and stable DNA structures, and they form central loop-stem type hairpins. Bulges or bubbles occur in the stem of an imperfect inverted repeat with one or more unpaired nucleotides. Spaced inverted repeats are common in the human genome and are often involved in disease-predisposed DNA rearrangements and gene amplification.^17^–^20^ In lower eukaryotes, such as *Tetrahymena*,^11^,^21^,^22^ *Schizosaccharomyces pombe*,^23^ and *Saccharomyces cerevisiae*,^12^,^19^ palindrome formation is mediated by short inverted repeats that naturally exist in the genome. Furthermore, artificial short inverted repeats can induce chromosome breaks and palindrome formation in *S. cerevisiae*,^19^ and engineered short inverted repeats constructs can mediate palindrome formation in mammalian cells following an adjacent DSB.^13^ Thus, palindrome formation is a critical step during BFB cycles, which might define the regions susceptible to gene amplification in cancer.^24^ Eventually, a chromosome starting with a DSB could end up with a repeated array of chromosomal segments, which would increase DNA rearrangements and lead to gene amplification in the form of HSRs and DMs.^25^

To better elucidate the molecular structure and amplification mechanism of DMs in ovarian cancer cells, DMs were dissected from the human ovarian cancer cell line UACC-1598. We identified two amplicons originating from 2p24.3 and 3q26.2 co-localized on the same DMs in UACC-1598. Moreover, a complicated 7 kb junction DNA fragment, which joined the two amplicons, was cloned and sequenced. Our sequence analysis has revealed *de novo* creation of small palindromic sequences surrounding the breakpoints is a common mechanism for definition of amplicon boundary and gene amplification. It also implicates that a novel DNA repair machinery may be involved in the creation of *de novo* small palindromic sequences identified in the DMs from ovarian cancer.

## Material and Methods

### Cell lines and reagents

Human ovarian cancer cell line UACC-1598, a cell line with spontaneously formed DMs, was obtained from the Tissue Culture Core Service of the University of Arizona Comprehensive Cancer Center. The cell line was maintained in RPMI-1640 medium supplemented with 10% fetal bovine serum^3^ (Invitrogen, Grand Island, NY). Spectrum Orange-dUTP and Green-dUTP were obtained from Vysis (Downers Grove, IL). BAC clones were from BACPAC Resources Center (Children's Hospital Oakland, Oakland, CA).

### Chromosome microdissection and degenerate oligonucleotide primed-polymerase chain reaction (DOP-PCR)

Metaphase spreads of UACC-1598 cells were prepared using standard procedures. Chromosome microdissection was performed as described previously.^26^ Briefly, 20 copies of DMs were dissected from UACC-1598 metaphase spreads. The dissected DMs were treated with Sequenase version 2.0 DNA polymerase (United States Biochemical Corporation, Cleveland, OH) and amplified by DOP-PCR with a degenerate primer widely used in the protocols of published literatures (5′-CCGACTCGAGNNNNNNATGTGG-3′).^26^

### FISH analysis

DNA probes were labeled with Spectrum Orange-dUTP or Green-dUTP, and then hybridized to metaphase spreads of UACC-1598 cells as described previously,^26^ and chromosomes were counterstained with 4,6-diamidino-2-phenylindole (DAPI). The high-quality metaphase images were captured using a Leica DM-RXA2 fluorescence microscope (Wetzlar, Germany), and analyzed using the MetaMorph Imaging System (Universal Imaging Corporation, West Chester, PA).

### Southern blot analysis

Ten micrograms of genomic DNA from UACC-1598 cells and the control cells (normal human peripheral blood leukocytes) were digested with *Eco*RI, fractionated on 0.8% agarose gel, transferred to a Hybond-N+ membrane and hybridized with ^32^P-dCTP-labeled probes. The probes were generated by PCR and the primers were listed in Supporting Information Table 1. Then the membrane was visualized on X-ray film by autoradiography.

### Construction of DMs DNA library and screening

The DNA from dissected DMs was cloned with an Advantage PCR Cloning Kit (Clontech Laboratories, Palo Alto, CA). Briefly, amplified microdissected DMs-specific DNA was subcloned into a pT-Adv vector, and the ligation products were transformed into TOP10F' competent *E. coli* (Invitrogen) and plated on LB-Amp/X-gal/IPTG agar plates. A total of 437 white colonies were randomly chosen and streaked. Colonies containing the highly repetitive sequences were identified by Southern blot hybridization using total human DNA probes. Positive clones randomly selected from the library were analyzed by DNA sequencing. The recombinant DNA sequences were aligned to the human genome sequence (hg19) at the University of California Santa Cruz (UCSC) Genome Bioinformatics website, and BLAT analysis was used to determine their chromosomal origins.

### Semi-quantitative and long-range PCR

Semi-quantitative and long-range PCR were performed to identify the precise boundaries and the junction sequences of the DMs amplicons. Genomic DNA from UACC-1598 cells and normal human peripheral blood leukocytes were extracted for these assays. For semi-quantitative PCR, the primers were designed on both ends of the two amplicons (AmpMYCN and AmpEIF5A2) (Supporting Information Tables 2 and 3), with *β-actin* as a control. PCR products were subjected to electrophoresis on agarose gel and semi-quantitatively analyzed by Alpha Inotech Imaging Systems and Fluochem Software (Alpha Inotech Corporation, San Leandro, CA). Long-range PCR was performed with the forward and the reverse primers which were designed at the distal ends of the amplicons (Supporting Information Tables 2 and 3). The orientation of the junctions was determined by long-range PCR. The detected long-range PCR products were purified and subcloned into the pGEM-T Easy Vector (Promega, Madison, WI) for DNA sequencing. These experiments were performed in triplicate.

### Web servers and bioinformatics tools

The human genome sequences (Build 37) were downloaded from the National Center for Biotechnology Information (NCBI). The sequence alignment to human genome was performed using the BLAST tool from NCBI and the BLAT tool from UCSC genome bioinformatics. Repeat sequence searching was performed using the RepeatMasker web server (http://www.repeatmasker.org). The DNA secondary structure analysis was performed using the mfold web server.^27^

## Results

### DMs in human ovarian cancer originate from 2p and 3q

**Figure 1:**
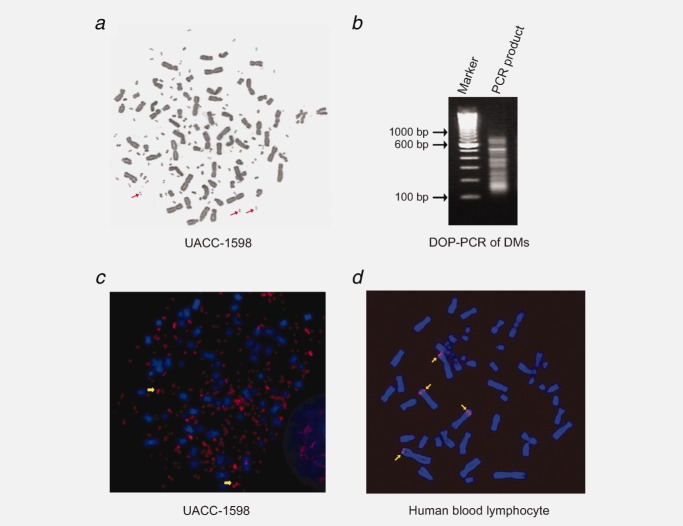
Chromosomal origins of DMs in UACC-1598 cells. (*a*) Karyotype of a UACC-1598 cell. Representative DMs are indicated by arrows. (*b*) DOP-PCR amplification of DMs. DMs isolated by microdissection were amplified and the PCR products were labeled as probes for following FISH analysis. (*c*) Representative image of FISH analysis in UACC-1598. Signals in red are mainly from DMs. Arrows indicate typical DMs. (*d*) Representative image of FISH analysis in a normal human peripheral blood lymphocyte confirms the DMs originate from chromosomes 2p and 3q.

To systematically study the DMs in human ovarian cancer, we investigated the chromosomal origin of DMs by mapping the DMs in the human ovarian UACC-1598 cancer cells (Fig. 1*a*). In this cell line, there are different DMs populations with the size of 1.4 Mb, 2.1 Mb and 2.8 Mb detected by pulsed field gel electrophoresis (PFGE) and Southern blot (Supporting Information Fig. 1). Chromosome microdissection was performed to isolate DMs. Twenty copies of DMs were isolated and amplified by DOP-PCR with a degenerate primer. The size of PCR products ranged from 100 bp to 1,000 bp (Fig. 1*b*). The DOP-PCR products were labeled with Spectrum Orange-dUTP, and hybridized back to the UACC-1598 metaphase spreads. The FISH analysis revealed that the labeled probes hybridized mainly to DMs (in addition to the original chromosome signals) (Fig. 1*c*), which demonstrated that the microdissected DNA was from the DMs. Then, the labeled probes were hybridized to normal human peripheral lymphocyte metaphase spreads to get their chromosomal origination information. Hybridization signals revealed that the DMs-specific probes specifically hybridized to 2p and 3q (Fig. 1*d*), and these results indicated that DMs in ovarian cancer UACC-1598 cells originate from chromosome regions 2p and 3q.

### Two amplicons in the DMs originates from the loci 2p24.3 and 3q26.2

**Figure 2:**
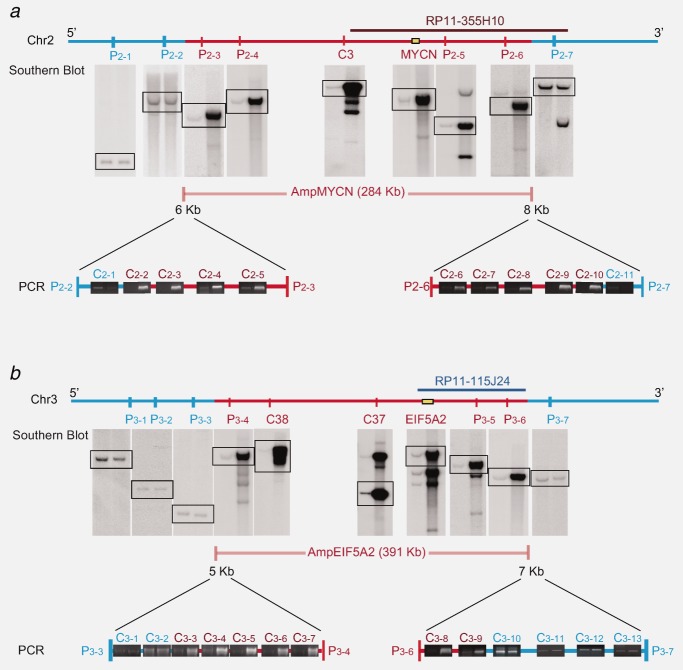
Mapping of the amplicons and their boundaries on chromosomes. Determination of the size and boundaries of AmpMYCN (*a*) and AmpEIF5A2 (*b*). The boundaries were narrowed to several kilobase by Southern blot and further narrowed to about 500 bp by semi-quantitative PCR. The loci in red indicate the regions amplified and the loci in blue are not amplified. Rectangles indicate the expected paired blotting results. For all paired results, the left lane was from control (normal human peripheral blood leukocytes DNA), the right lane was from UACC-1598 cells.

To determine the exact location of DMs on chromosomes 2p and 3q, a DNA library composed of DOP-PCR products was constructed. After library screening, DNA sequencing was performed to confirm the accurate chromosomal origins of the DMs. These results revealed that clone C3 shared significant sequence homology with the sequence at locus 2p24.3, next to the *MYCN* gene (Fig. 2*a*), while the other two clones, C37 and C38, shared significant sequence homology with the sequence at locus 3q26.2, next to the *EIF5A2* gene (Fig. 2*b*). Southern blot using the three clones as probes confirmed that these fragments were remarkably amplified in the DNA of UACC-1598 cells compared with normal cells (Figs. 2*a* and 2*b*). Therefore, DMs in cell line UACC-1598 bear amplicons originated from the loci 2p24.3 and 3q26.2.

To determine the accurate boundaries and sizes of these amplicons at loci 2p24.3 and 3q26.2, primers for *MYCN*, *EIF5A2* genes and 14 surrounding loci were designed, and the corresponding PCR products were used as probes for Southern blot hybridization. The amplification level of the probe in UACC-1598 was measured by using normal human DNA as a control. With a series of analyses, a rough map of the amplicon boundaries was determined (Figs. 2*a* and 2*b*). Noteworthily, multiple additional fragments were found for probes P_2–5_, P_2–6_, P_3–4_ and P_3–5_ in UACC-1598 compared with the control DNA. Then, a series of PCR primers were designed to determine the precise loci of breakpoints for the boundaries of amplicons. With a series of semi-quantitative PCR assays, breakpoints were defined at each boundary of the amplicons in a range less than 500 bp (Figs. 2*a* and 2*b*). By using BLAT to align primer sequences with the reference human genome sequences, we determined that the amplicon at locus 2p24 was about 284 kb and the amplicon at locus 3q26 was about 391 kb (Figs. 2*a* and 2*b*), referred to as AmpMYCN and AmpEIF5A2, respectively.

### DMs are formed from two amplicons joined by a complex junction

**Figure 3:**
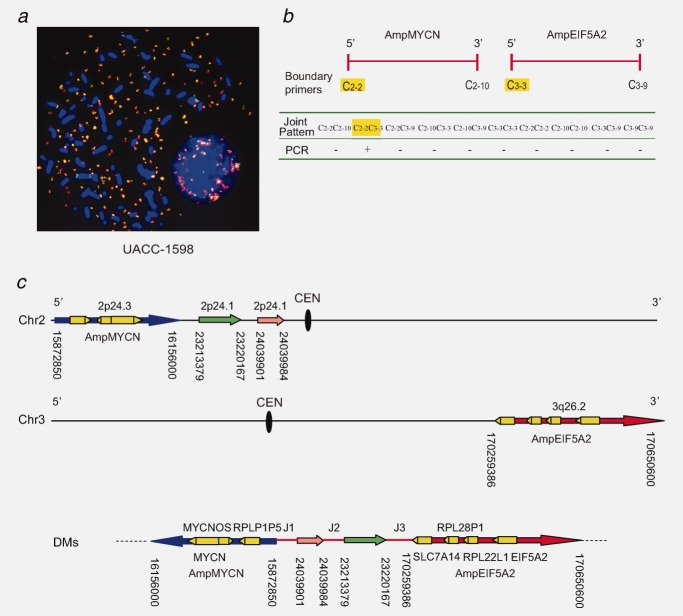
Identification of the molecular structure of DMs. (*a*) AmpMYCN and AmpEIF5A2 co-localized on the same DMs. The merged signals proved the co-localization. (*b*) Determination of the joint pattern of AmpMYCN and AmpEIF5A2. Possible joint patterns were determined by long-range PCR using primer pairs designed. (*c*) The molecular structure of DMs in UACC-1598 cells. AmpMYCN (blue) from 2p24.3 and AmpEIF5A2 (red) from 3q26.2, are linked by two discontinuous 2p24.1-originating fragments with a size of 84 bp (pink) and 6789 bp (green). The junction parts, J1, J2, J3, are shown as red lines. The yellow triangle squares represent genes on the DMs.

To further analyze the amplicons, two color FISH was performed with probes RP11–355H10 and RP11–115J24 (BACs specific to AmpMYCN and AmpEIF5A2, respectively) hybridized to UACC-1598 cells. Results showed that AmpMYCN and AmpEIF5A2 co-localized on the same DMs (Fig. 3*a*).

The co-localization result also suggests that the two amplicons (AmpMYCN and AmpEIF5A2) might be joined together. In theory, ten possible joint patterns may exist in between these borders (Fig. 3*b*). To clone all possible junction sequences, we applied long-range PCR methods using forward (C_2–2_, C_3–3_) and reverse (C_2–10_, C_3–9_) primers orienting toward the breakpoints, selected from the previous border primers. The results showed that a DMs junction with the C_2–2_–C_3–3_ joint pattern was successfully amplified and cloned (Fig. 3*b*). This PCR product suggests that both 5′ ends of AmpMYCN and AmpEIF5A2 are joined together. The joint fragment, about 7 kb in size, was cloned and sequenced. The result confirmed the rejoining pattern of AmpMYCN and AmpEIF5A2 at the sequence level. In detail, two discontinuous fragments, 84 bp and 6,789 bp in size, originating from 2p24.1 (chr 2: 24039901–24039984; chr 2: 23213379–23220167) are present in the junction and join the AmpMYCN and AmpEIF5A2 amplicons. As shown in Figure 3*c*, the rearrangements among the 2p24.3-, 2p24.1- and 3q26.2-originating fragments include three breakpoint-rejoining events: (1) the 5′ end of AmpMYCN (chr 2: 15872850) is rejoined to the 5′ end of the 84 bp fragment (chr 2: 24039901), (2) the 3′ end of the 84 bp fragment (chr 2: 24039984) is rejoined to the 5′ end of the 6,789 bp fragment (chr 2: 23213379) and (3) the 3′ end of the 6789 bp fragment (chr 2: 23220167) is rejoined to the 5′ end of AmpEIF5A2 (chr 3: 170259386).

In conclusion, the non-syntenic genetic loci of AmpMYCN (284 kb) and AmpEIF5A2 (391 kb) connected by a 7 kb complex junction DNA sequence comprise the core structure of DMs. In addition to *MYCN* and *EIF5A2*, three other genes *MYCNOS*, *RPL22L1* and *SLC7A14* and two pseudogenes *RPLP1P5* and *RPL28P1* are also present on the DMs. Among the DMs bearing genes, *SLC7A14* has a 3′-end truncation (Fig. 3*c*).

### *De novo* created small palindromic sequences are identified in between amplicon boundaries

**Figure 4:**
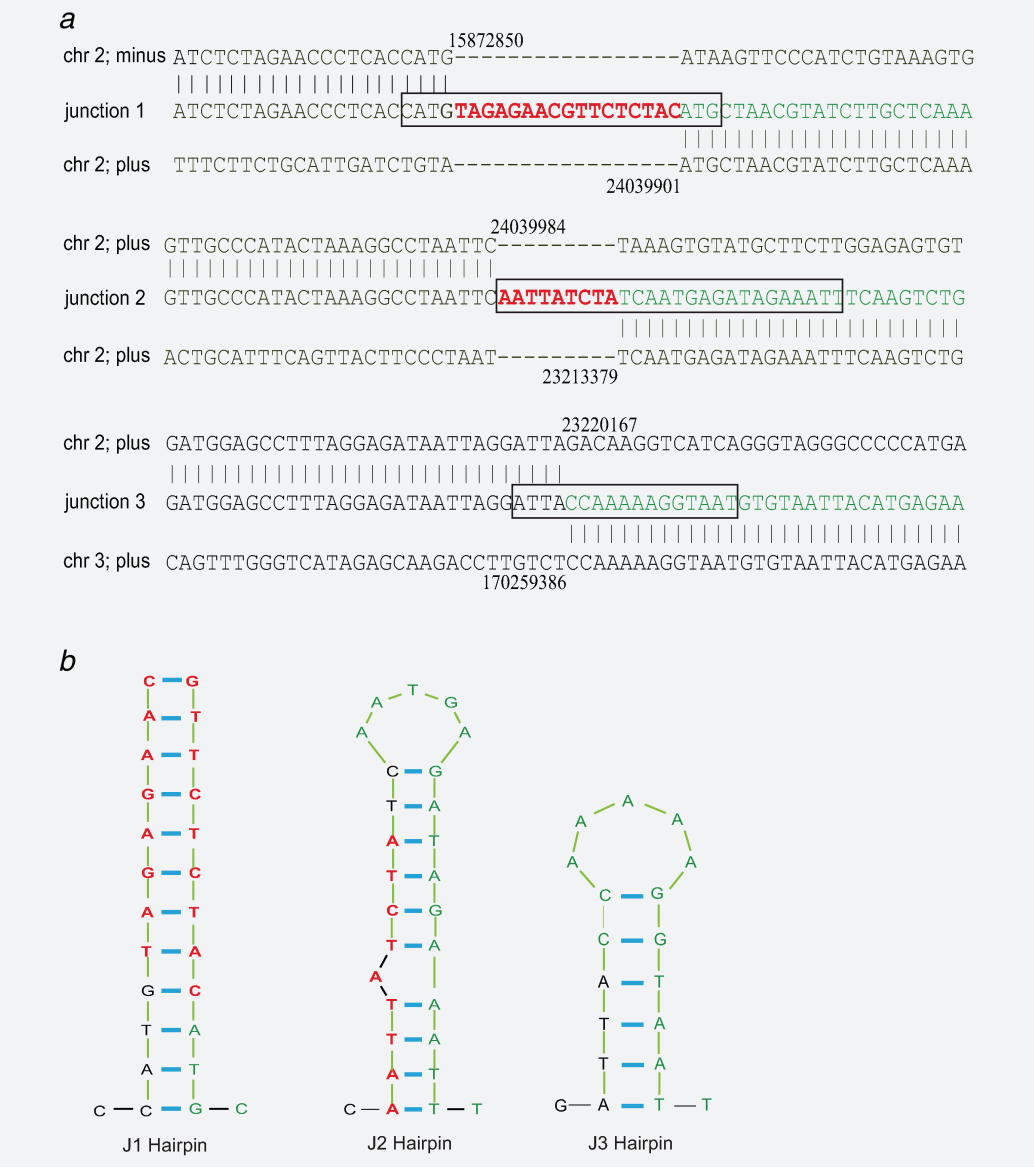
Sequences and *de novo* palindrome structures of junctions on DMs. (*a*) Breakpoints and junction sequences of DMs. Two artificial insertions, 17 bp in Junction 1 and 9 bp in Junction 2, were identified (red). Junction 3 is formed by blunt-end joining. Natural sequences from different chromosomes are depicted either in black or in green. Palindromic sequences are depicted in black rectangles. (*b*) *De novo* palindromic structures occur in all three junctions. The predicted stem-loop type hairpin structures are illustrated.

On the DNA sequencing results, we obtained six breakpoints that are joined by three junction sequences as shown in Figure 4*a*. These joined sequences are non-syntenic (chr 2 and chr 3) or syntenic but discontinuous (2p24.3 or 2p24.1). In Junction 1 and Junction 2, a 17 bp and a 9 bp insertion were found, respectively, (Fig. 4*a*). Junction 3 is formed by blunt-end joining.

To reveal the characteristic sequence that participates in junction formation, we did analysis into the non-homologous junction sequences. As homologous recombination (HR) may mediate rejoining by repetitive sequences, we searched for repetitive sequences within the 2 kb regions on both sides of each breakpoint using the RepeatMasker web server. In Junction 1 and Junction 2, none of the breakpoints were located in repetitive sequences. Both breakpoints of Junction 3 were located in repetitive sequences, but of the different types (Supporting Information Fig. 2). Thus, the rejoining of these non-homologous broken ends is not mediated by HR.

Most strikingly, three small palindromic structures were found in the junctions (Fig. 4*b*). In Junction 1, a small perfect palindrome which can form a 24 nt stem-type hairpin is found. Junction 2 has a palindrome which can form the hairpin structure with a 20 nt stem, 5 nt loop and one unmatched nucleotide. In Junction 3, a palindrome forming the hairpin with a 12 nt stem and 5 nt loop is also predicted. Furthermore, the two insertions participate in the stem formation of the hairpins in both the Junction 1 and Junction 2. The Junction 3 palindrome is composed of sequences originating from different chromosomes (Chromosome 2 and Chromosome 3). Therefore, all three small palindromes located in the boundary and rejoining the amplicons are *de novo* created, and do not naturally exist in the human genome.

## Discussion

DMs, one of the cytogenetic manifestations of gene amplification, have been observed in most solid tumors and many hematological malignancies. The presence of DMs in cancer patients is correlated with poor prognosis and poor chemotherapeutic response.^28^–^30^ Many chromosomal regions, such as 8q24, 3q26, 7p12, 16q, 22q23, 17q21, 1q21, 12q14–15, 4q12 and 7q31, carrying oncogenic genes, including *MYC*, *EGFR*, *MYCN*, *EIF5A2*, *ATBF1*, *MDM2*, *DDX1*, *ERBB2*, *COAS*, *GLI*, *PDGFRA*, *MET* and *TRIB1*, have been reported to be amplified on DMs in human cancers.^31^–^35^ Co-amplification of different oncogenes or chromosomal regions was also reported in human cancers. Four extra-chromosomally amplified loci were reported in a glioma,^36^ and co-amplification of syntenic but discontinuous or non-syntenic segments were found in neuroblastoma and small cell lung cancer (SCLC) cell lines.^36^ Non-syntenic co-amplification is also reported in hematological malignancies.^37^ However, the detailed molecular structure of DMs is largely unknown. Studies trying to elucidate the architecture of DMs, in particular those with complex amplicon structure are limited.^35^,^36^,^38^–^40^

In human ovarian cancer cell line UACC-1598, the 3q26 amplicon was previously identified on DMs and a proliferation-related function of *EIF5A2* gene was reported.^3^ In this study, we further identified that 2p24.3 and 3q26.2 were the origins of DMs in UACC-1598, and genes including *MYCN* and *EIF5A2*, *MYCNOS*, *RPL22L1*, *SLC7A14* (3′-end truncated) and two pseudogenes *RPLP1P5* and *RPL28P1* were co-amplified on the same ovarian DMs. We have determined the precise breakpoints of the AmpMYCN and AmpEIF5A2 amplicons and cloned the complex 7 kb sequence joining the two amplicons. The joint sequence originates from 2p24.1, and is composed of two discontinuous fragments with complex rearrangements.

The junction sequence analysis can give us clues for the underlying rejoining mechanism. The rejoined non-homologous broken ends are not located in the same type of repetitive sequences excluding the possibility of HR rejoining mechanisms. Instead, the non-homologous end joining (NHEJ) mechanism is more likely to be involved in DNA rejoining with junctions showing the features of small insertions and blunt-end joining. Previous reports on DMs junction are in concert with our findings which suggested the NHEJ mechanism is responsible for the rejoining of the broken ends with the junctions showing the features of microhomologies, insertions or blunt-end joining.^35^,^36^,^38^ However, are there some more common characteristics in the DMs junctions? Interestingly, we found three small *de novo* palindromes surrounding the breakpoints in the joint sequence. In Junction 1 and Junction 2, the palindromes were formed after the rejoining and insertion. In Junction 3, the palindrome is formed after the rejoining of two non-syntenic sequences. Further, we analyzed the DNA sequences of 57 previously reported junctions on DMs from gliomas,^38^ neuroblastomas, SCLC^40^ and hematological malignancies.^35^ *De novo*-generated short hairpin structures surrounding the breakpoints with a stem which has ≥4 bp matched nucleotides were identified in about 70% (39 of 57) of these junctions. Moreover, the illegitimately joined broken ends (13 of 39 with blunt-end joining, 7 of 39 with microhomology), as well as the *de novo* insertions (19 of 39), take part in the formation of the palindromes (details are summarized in Supporting Information Fig. 3 and Supporting Information Table 4). Taken together, our results suggest that *de novo* creation of small palindromes may be a prevalent characteristic of boundary definition and amplicon rejoining.

To date, one of the underlying amplification mechanism is DNA DSB triggered palindrome formation.^41^–^43^ A non-random distribution of palindromes in cancer cells might serve as precursors for gene amplification.^24^ Regarding to the DMs in UACC-1598 cells and other reports, small junction palindromes are created *de novo*, which are in sharp contrast with previous findings from *T. thermophila*,^11^ *S. pombe*,^23^ and *S. cerevisiae*,^19^ in which naturally existing inverted repeats form hairpins following DSBs, define the gene amplification boundary. Combining our observations with previous findings, we suggest that the unknown mechanism for palindrome *de novo* synthesis and palindrome surveillance might function in concert with NHEJ to generate the palindrome-containing junctions which promote gene amplification (Supporting Information Fig. 4). We speculate that the mechanism must confer at least two activities. The first is surveillance activity to identify fortuitous palindromic sequences surrounding the two break ends that will be joined together. Like the case of the palindrome in Junction 3, a fortuitous palindromic sequence exists on both break ends; thus, Junction 3 is formed by blunt-end joining involving MRN (Mre11/Rad50/Nbs1), DNA-PK, Ku70/80 and Lig IV/XRCC4 DNA repair components of the NHEJ machinery (Supporting Information Fig. 4*a*). The second activity is *de novo* synthesis to create a novel palindromic sequence in between the two break ends. Once the speculated surveillance enzyme determines that there is no short inverted repeat on both ends, a class of DNA polymerases is recruited to the 3′ ends of the two breaks to extend each end by adding non-templated nucleotides to synthesize *de novo* palindromes (Supporting Information Fig. 4*b*). Alternatively, this surveillance enzyme may itself serve as the enzyme for creating the palindromes. Eventually, by annealing and ligating the two extended fragments, possibly through Lig IV and XRCC4 from NHEJ, the rejoined DNA segment possessing newly created palindromes would go through a DSB induced, short inverted repeats mediated canonical gene amplification. In the NHEJ pathway, DNA polymerase X family has a gradient of template dependency. For example, terminal deoxynucleotidyl transferase (TdT) is a template-independent polymerase adding random nucleotides (called N nucleotides) to the junction.^44^,^45^ Polymerases mu and lambda can generate inverted repeats (called T nucleotides) in the junction by the flexible use of nearby sequences as templates.^46^ So the polymerases, the nuclease trimming the DNA ends, as well as the other enzymes with potential surveillance functions, can be involved in the mechanism we propose for the *de novo* generation of the small palindromes in the junctions. The small palindromes can provide the platform for amplification, followed by DSBs and BFB cycles. From Southern blot analysis, we found multiple fragments for some probes which were near the junctions (such as probes P2–6, P3–4 and P3–5) in the UACC-1598 DNA compared with the control DNA. We suggest that the small palindromes in the junctions may trigger further rearrangements and generate the additional fragments for these probes.

In conclusion, the core structure of DMs in human ovarian cancer cell line UACC-1598 is constructed and analyzed in detail. Differently originating amplicons co-amplified on DMs with high complexity. Our findings demonstrate that *de novo* creation of palindromic sequences surrounding the boundary breakpoints is prevalent at the DMs junctions and may play a common and pivotal role in mediating palindromic amplification in human cancer cells.

## What's new?

Double minutes (DMs) are poorly understood hallmarks of gene amplification, a form of genomic aberration associated with tumor development. After cloning DMs from a human ovarian cancer cell line, the authors determined the precise breakpoints of two co-amplified amplicons and analyzed the complex DNA sequence joining them. They found that *de novo* creation of small palindromic sequences surrounding the breakpoints is a common characteristic of amplicon junction sequences in cancers. The palindromic sequences, which may be generated through non-homologous end joining in concert with a novel DNA repair machinery, may play a common role in amplicon rejoining and gene amplification.

## Abbreviations

BFB: breakage-fusion-bridge
CHO: Chinese hamster ovary
DAPI: 4,6-diamidino-2-phenylindole
DMs: double minutes
DOP: degenerated oligonucleotide primed
DSBs: double-strand breaks
HR: homologous recombination
HSR: homogeneously staining region
NCBI: National Center for Biotechnology Information
NHEJ: non-homologous end joining
PCR: polymerase chain reaction
PFGE: Pulsed field gel electrophoresis
SCLC: small cell lung cancer
UCSC: University of California Santa Cruz
