# Supplementary material for: De novo-generated small palindromes are characteristic of amplicon boundary junction of double minutes
Source: Int J Cancer. 2013 Mar 9;133(4):797–806. doi: 10.1002/ijc.28084 (PMC3734650; doi:10.1002/ijc.28084)

Supplementary Table 1. PCR primers for the probes of Southern blot

Probe	Sequence	Product Size (bp)
P_{2-1}-F	TCTGTATCCTTTCCCTCTTGG	516
P_{2-1}-R	GCAGCCCATCTTTGTGTG	
P_{2-2}-F	TGCCATCTATTGCTCTCTGG	525
P_{2-2}-R	TAGCTCCAAACTGAGGGTGT	
P_{2-3}-F	GTAAGTATTCTACATGCCTGAG	400
P_{2-3}-R	ATGCTACTGGGTTAACATC	
P_{2-4}-F	GCTGTCTAGGGCACCAATC	515
P_{2-4}-R	CTCTCTGCTGGGATGTGTCT	
P_{2-5}-F	CCCTCTCCCTCAAACATCTC	473
P_{2-5}-R	TCCTACCCCTCAAATGAAGAC	
P_{2-6}-F	CCAAATGTGCCCTAAATCC	489
P_{2-6}-R	TGAGTAACATTCCCTGCTGA	
P_{2-7}-F	GGGGACCTCTGTACTCAAAA	476
P_{2-7}-R	AAGGAACCTGGCACTGAAA	
P_{3-1}-F	TTGCCCCTGAGTTACCTTTC	499
P_{3-1}-R	ACCTTGTCCCAACCCACTTT	
P_{3-2}-F	TGGGTCCTTTGGAAATAGAG	489
P_{3-2}-R	GAGGCAGGATGGTTTACAGA	
P_{3-3}-F	GCCTCAGTCTTTTACCCAATC	540
P_{3-3}-R	TGTTCCTTACCTGGCTCTATG	
P_{3-4}-F	CCTCTGCTGCCCTTTAATGA	494
P_{3-4}-R	GCTTTACTGGCATGGGTCAA	
P_{3-5}-F	AGCGTTTACAGAGGATTTGG	483
P_{3-5}-R	GAGCCAAGATGGGATAGAAG	
P_{3-6}-F	GCCATGGTGGAGGTCTTATT	316
P_{3-6}-R	GGAGGGAAAGCATTCAAAC	
P_{3-7}-F	TAATCAGGGGATGGGAATCT	307
P_{3-7}-R	CCTACTGGCTGTCTTATACTCACT	
MYCN-F	AAGGCTGTCACCACATTCAC	596
MYCN-R	CCGAGCGTGTTCAATTTTC	
EIF5A2-F	ATGAACGGATCCACCATGGCAGACG	505
EIF5A2-R	CTAAGACGAATTCAGACATAAACAGTGTTCATG	

Supplementary Table 2. Primers used to determine the boundaries of AmpMYCN

Primer	Sequence
$C_{2\text{-}1}$-F	CATCAGCCTTCTGTTACCTG
$C_{2\text{-}1}$-R	TCAGTGACGACAGAGAAAGG
$C_{2\text{-}2}$-F	ACAGCTCAGGAGACGTAATC
$C_{2\text{-}2}$-R*	AGCCTCAAGAGATACACTGG
$C_{2\text{-}3}$-F	ATCATGGTGAGGGTTCTAGAG
$C_{2\text{-}3}$-R	TTTCTCTCATCCAAGCGTC
$C_{2\text{-}4}$-F	TCCTGACCTTGTGATCTGCC
$C_{2\text{-}4}$-R	GACATAAAGGTTCCAATCGAGC
$C_{2\text{-}5}$-F	TCCTGACAGATTTGAGTCTCTC
$C_{2\text{-}5}$-R	GAGTTGTGTAGTCATGTCTAGCC
$C_{2\text{-}6}$-F	CCAAATGTGCCCTAAATCC
$C_{2\text{-}6}$-R	TGAGTAACATTCCCTGCTGA
$C_{2\text{-}7}$-F	CTGCCTTTAACTTGCTACTGTC
$C_{2\text{-}7}$-R	GGAGCCTCATCTTGTTCATC
$C_{2\text{-}8}$-F	GCCAATGAACTCAAGACTAGTG
$C_{2\text{-}8}$-R	ACCTGCAGTGAATGAGAAGAG
$C_{2\text{-}9}$-F	AACTTTGCCAAGAGCTCAG
$C_{2\text{-}9}$-R	ATGCTCTGCCTATTGCTAAG
$C_{2\text{-}10}$-F*	GCTTCTGCTGATTCCTCC
$C_{2\text{-}10}$-R	GTTCAGCAAGCGTGAGTG
$C_{2\text{-}11}$-F	GTTCCCACTTCCTAGTCACTTC
$C_{2\text{-}11}$-R	ATTGCCTGAGACTAGCTTGTG
β-actin-F	CTCCTCAGATCATTGCTCCT
β-actin-R	TCACCTTCACCGTTCCAGTTTT

*Primers for long-range PCR (all primers were used for semi-quantitative PCR).

Supplementary Table 3. Primers used to determine the boundaries of AmpEIF5A2

Primer	Sequence
$C_{3\text{-}1}$-F	GCCTCAGTCTTTTACCCAATC
$C_{3\text{-}1}$-R	TGTTCCTTACCTGGCTCTATG
$C_{3\text{-}2}$-F	GCTGACACAGGAGGATTGCTTG
$C_{3\text{-}2}$-R	TGGCTCCTGGTGATTCGATTC
$C_{3\text{-}3}$-F	CAGCAGAATCGAATCACCAG
$C_{3\text{-}3}$-R*	GGCCAAGTGAGAGAGATGAAG
$C_{3\text{-}4}$-F	TGTCCCTTGCCACTACAGCTCC
$C_{3\text{-}4}$-R	GGCATCATGGCAAACTGGATTC
$C_{3\text{-}5}$-F	GTATGCAGAGGACAGTGTGAG
$C_{3\text{-}5}$-R	GCTTCTTAAAGACAGAGGGAC
$C_{3\text{-}6}$-F	AAGAACCACAGTCTCTCCAG
$C_{3\text{-}6}$-R	TAAGCACGCTCAATACTAGG
$C_{3\text{-}7}$-F	CCTCTGCTGCCCTTTAATGA
$C_{3\text{-}7}$-R	GCTTTACTGGCATGGGTCAA
$C_{3\text{-}8}$-F	GCCATGGTGGAGGTCTTATT
$C_{3\text{-}8}$-R	GGAGGGAAAGCATTCAAAC
$C_{3\text{-}9}$-F*	AAAAAGCCCAACAGTGAACA
$C_{3\text{-}9}$-R	AACCAACCCAAATGTCCATC
$C_{3\text{-}10}$-F	TCTTCTTGCCCAGGATTGTC
$C_{3\text{-}10}$-R	GATGCCCTCTCTCACCACTC
$C_{3\text{-}11}$-F	ACTGCTTTGAATGTGTCCCAG
$C_{3\text{-}11}$-R	AGACACAGACTGGCAAATTGG
$C_{3\text{-}12}$-F	CACACTGATGGGTCTTGACTC
$C_{3\text{-}12}$-R	TCTCGGCAGAAACTCTACAAG
$C_{3\text{-}13}$-F	GAGTCTACAGAGGCTGGCAG
$C_{3\text{-}13}$-R	CAGCATGAGCAACACAGAAG
β-actin-F	CTCCTCAGATCATTGCTCCT
β-actin-R	TCACCTTCACCGTTCCAGTTTT

*Primers for long-range PCR (all primers were used for semi-quantitative PCR).

Supplementary Table 4. Summary of structure analysis of 57 published junctions in DMs from human tumors

Tumor type	No. of junctions	No. of de novo hairpins[a]	No. of artificial insertion hairpins[b]	No. of blunt-end joining hairpins[c]	No. of microhomology joining hairpins[d]
Hematological malignancy	5	5	2	1	2
Glioma	20	14	7	4	2
Neuroblastoma	24	15	5	6	3
SCLC[e]	8	7	5	2	0
**Total**	**57**	**39**	**19**	**13**	**7**

[a] These are hairpin structures artificially generated from rejoining of origin-different sequences with the characteristics of small origin-unknown insertions, blunt-end joining, or microhomology.
[b] These are de novo hairpin structures with the insertions composed or at least partially composed of the stems of the resulting hairpins.
[c] These are de novo hairpin structures resulting from blunt-end joining of segments with different chromosomal origins.
[d] These are de novo hairpin structures resulting from two segments with different chromosomal origins rejoined by a microhomology mechanism.
[e] Small cell lung cancer.

Supplementary Figure 1. Molecular size of DMs

This file provides the analysis results of the molecular size of DMs by PFGE and Southern blot. (A) Separation of DMs with PFGE. Linearized DMs were separated on a 0.8% agarose gel in 1 x TAE in a PFGE apparatus, and recirculated at 14℃. The run time was 48 h at 3 V/cm with 500 s switch time ramp at an included angle of 106. (B) Southern blot hybridization of DMs with EIF5A2 gene probe. By X-ray treatment or *Not*I digestion for 60 min, the southern blot results showed consistent bands with the size of 2.8 Mb, 2.1 Mb and 1.4 Mb respectively. The small-sized molecules seen in the *Not*I digestion for 60 min line are incompletely-digested products. The smear bands in the front of the gel on both the X ray line and the *Not*I digestion for 30 min line are small fragments of nonspecific digestion of DMs. M indicates the marker H. wingei. X indicates DMs were linearized with X-ray; N1 indicates DMs were linearized with *Not*I digestion for 30 min; N2 indicates DMs were linearized with *Not*I digestion for 60 min.

Supplementary Figure 2. Repetitive sequences in the vicinity of each breakpoint

The repetitive sequences (blue rectangles) appeared 2 Kb upstream or downstream of the breakpoints. The names of the repetitive sequences are indicated under the blue boxes. The solid horizontal line indicates the amplified sequences, and the dotted horizontal line indicates the non-amplified region. The red vertical line indicates the breakpoint sites. The figure is plotted according to the UCSC genome browser (hg19).

Supplementary Figure 3. Analysis of the reported DMs junction sequences which can form *de novo* palindromes and hairpin structures

The 39 junction sequences identified in human tumors were presented, including their *de novo* generated palindrome structure after rejoining. (A) Hematological malignancies. (B) Gliomas. (C) Neuroblastomas. (D) Small cell lung cancer (SCLC). The differently originated sequences are in black or green, the small insertions are in red, and the microhomologies in pink.

Supplementary Figure 4. Model of de novo creation of small boundary palindromes.

We propose that the unknown mechanism for palindrome de novo synthesis and palindrome surveillance might function in concert with NHEJ to generate the palindrome-containing junctions. (A) In the surveillance pathway, during the rejoining of two DNA segments, the unknown surveillance enzyme (pink Survey oval) could detect fortuitous palindromic sequences existing at

the end of a broken strand. If a potential palindrome (rectangle with black arrow) detected at both ends, the two broken strands would be joined by blunt-end joining through NHEJ-mediated DNA repair. (B) In the de novo synthesis pathway, under the condition that a DSB occurs on a region without any inverted repeats, the speculated surveillance enzyme recruits a non-templated DNA polymerase at the 3′ end of each break, and initiates de novo synthesis of palindromic sequences (dotted pink lines). By annealing and ligating the two extended fragments, possibly through Lig IV and XRCC4 of the NHEJ DNA repair mechanism, the rejoined DNA segment possessing newly created small palindromes (rectangles with green or yellow arrows).

Supplementary Fig.1

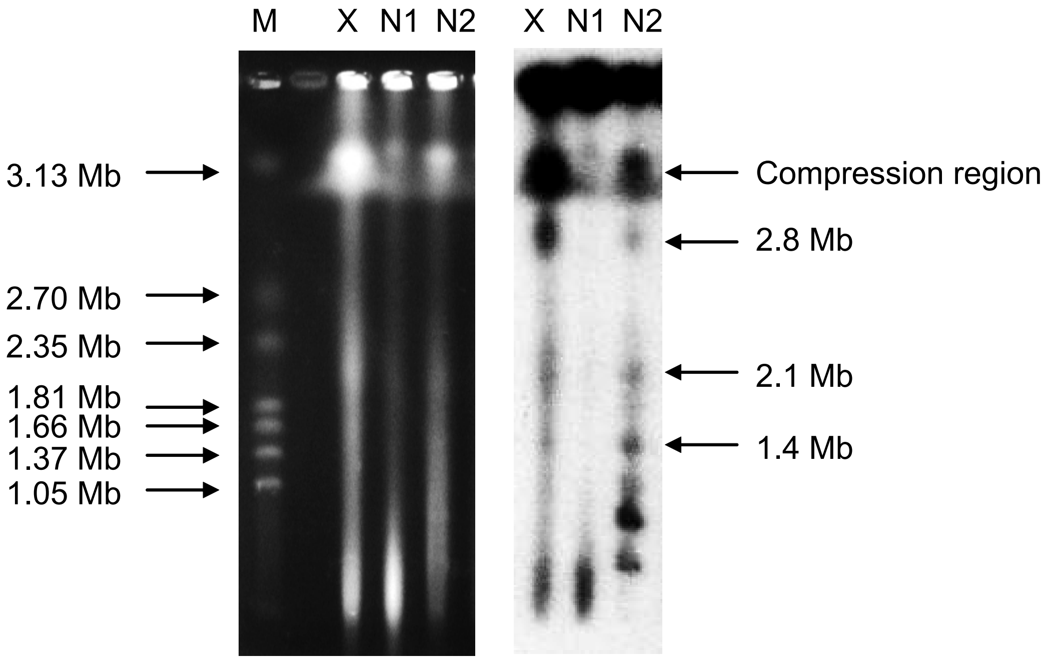

Supplementary Fig.2

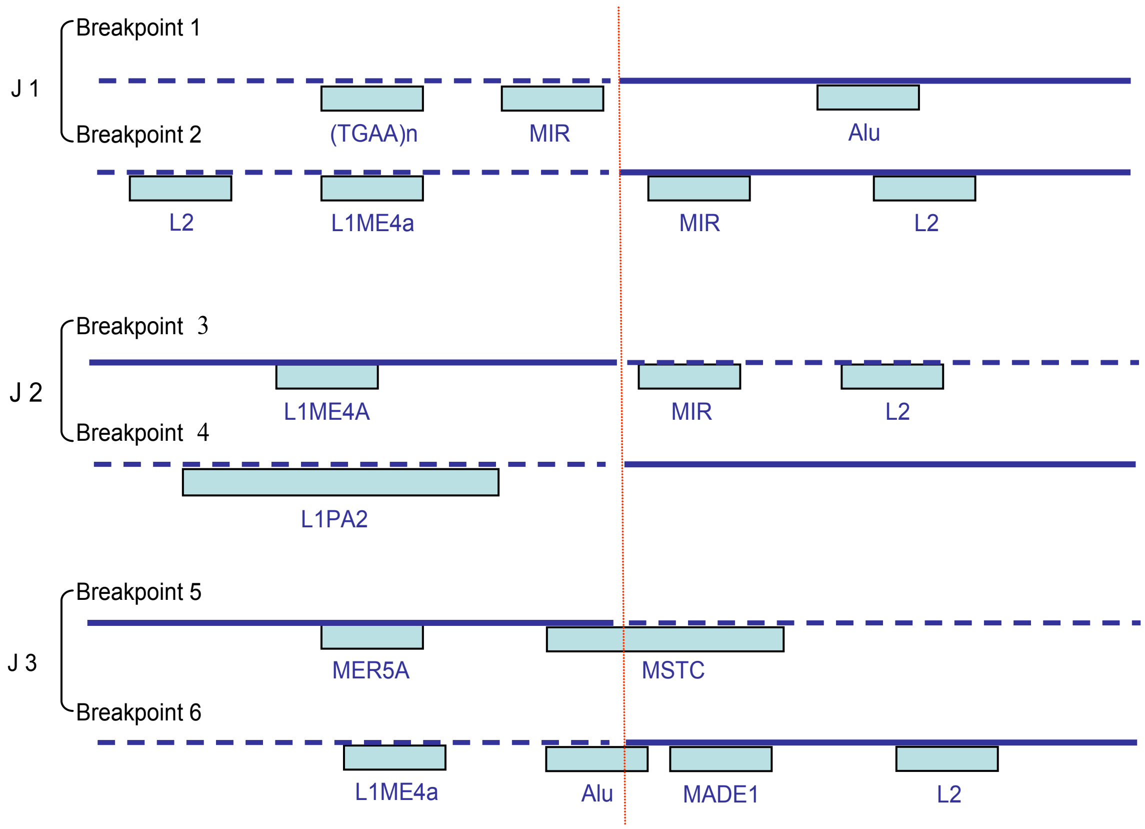

Supplementary Fig.3

A. Hematological malignancies

1 CAGGGATGTCCATACCTGCGTGTCACCGGAGCAGCTGGGACTACAGTT

2 GGTGGTACCCCCATTCTGTGCTCATTCTGGTACCCCCATTCTGTAGCTCTCAGTCCTGC

3 AGTTCCTTTGACCAAGGAGCCCTCCTCATGGTAATTAGAAACTCTGATT

4 CTATTTTTCAAAATAATAGCCCCAGTAGTAAGACTGGTTGTTATTTTT

5 CTTAAGGACTCAGGCCTAAACCATTTGTTGACTAAATGAATACTGTTC

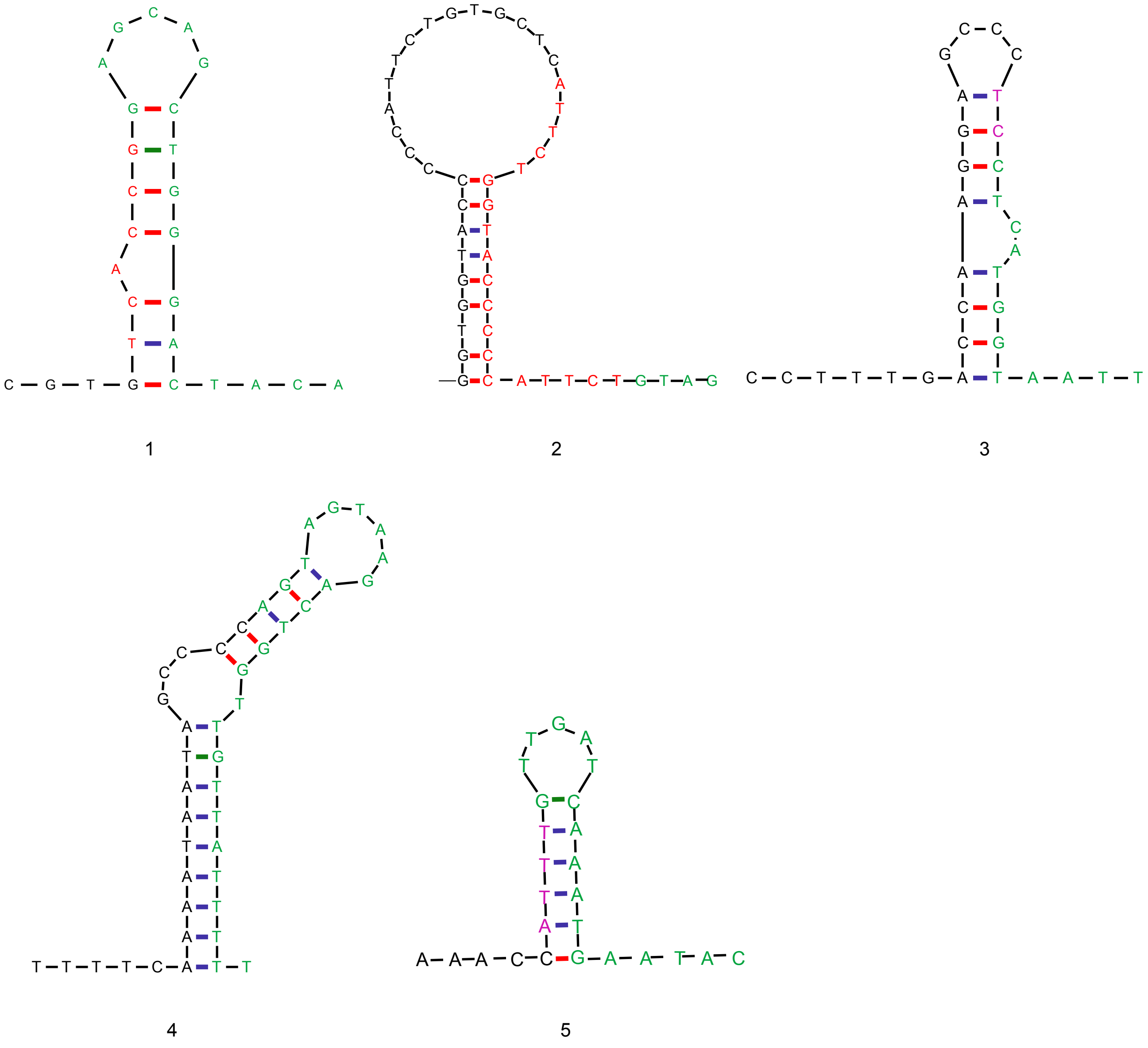

Supplementary Fig.3

B. Gliomas

1 TTTATTTATGTTTTTGTTTTGAGACCAACAACACACTTGGTTGTC

2 TCTGTGCAGCATGAAGTAGCCTGATACTGATACTGGCCTGATAGA

3 CTGTGATTTGATAATGTGCCACTTACCCATCAGGCACTTTTCATA

4 ATTAGCTGGGCATGGTGGCAGGCACAATTTAGTATACTCCTATGA

5 GGAAGGAGCCCAGTTCCTGCGAAGGGTCGGGGAGGAAGGG

6 CTTTTTCATGCTGTTCTCGTGGGGTAATCAGATACGCGGCGC

7 AAACACAGTTTACTTTAGAGCTGTAACAAGGTAACAAGT

8 CACTGCAGGTAAAAATGATGAGTGTAAGGGTTAATGGCCCT

9 GGAGGATGCCTCGGGGCGTCGCACAATTGCCTGCATGTCTA

10 AAATATAAAAGTCAGAATATAGACTCCACCTCAAAAAAAAAAA

11 TCACTGGGACTGGTTAGACAGTTCAGGTGATCTGCCCGCCCTGGC

12 GGAGCGATCATTGCTCACTGGCCAGAGGTCTGCAGACAGA

13 TCCCTTGAGAAGGGTGCTTCCTTTAGAGGCACCGTGGGACCAA

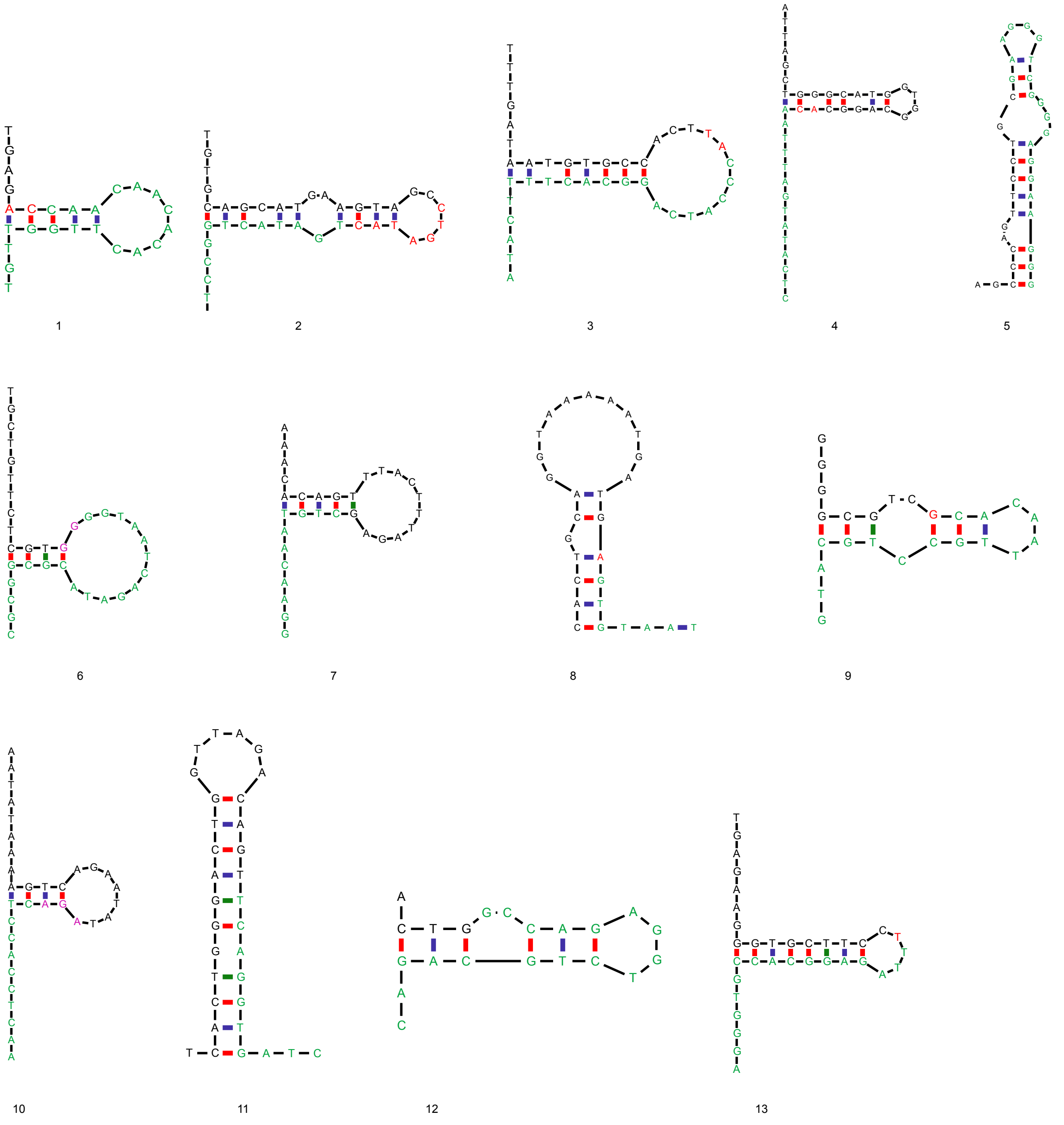

Supplementary Fig.3

C. Neuroblastomas

1 TGAGTTGCAGGAGAATTCTGTACTAATTAATTAGTACCTCATTCTACATTTATAATTTCTATATAACAAGGGACCCCGCTG

2 CGATGAATCGAAATTTATTTTTTATTGGTATTTTTAGCAGTGTGGCGA

3 TTGTATCTTCATAGCCAAATGTGGTTCTGGCCCCTGTTCTG

4 TCTTTGTGTGTGTGTACGCACTCCTTCTATTGGCGTTGAA

5 TATGTGTCCATGTGTCCATAACATCGCCTTCCTAGTGCTCAGTAAATATTTCAAGACTTTCTTGA

6 GCAAGAGTGAAGATGTGTGGTTAGAGTCTATTTCTCATCAT

7 TGTGTATGGGAGCAACTCACTAACCGGACTCGTCTCGCAA

8 TTCAAGCTGGCTGCGGAAATACAAGAATTCACTTTCATCTT

9 AAAGAAGAAAAGAGAGAAGATCACCAAACATTTTCTACACA

10 TTGGTAAATGTGCTAAGTTGTGCACCCACCTTGGCCTCCCAAA

11 TGTTTCCGATGAATCGAAATTTATTTTTTATTGGTATTTTTAGCAGTGTGGCGATTCC

12 CTCTATTATTTTCATTGACAACTGTGATGAGGGCAGGGCC

13 ATATAAAGGATCTGAAGGGTGCTGCCGGTGAGCGAGGCCAC

14 TCAAAACACCTGTAAGCCAGCTTCGGCTCATTTGCAGTCAT

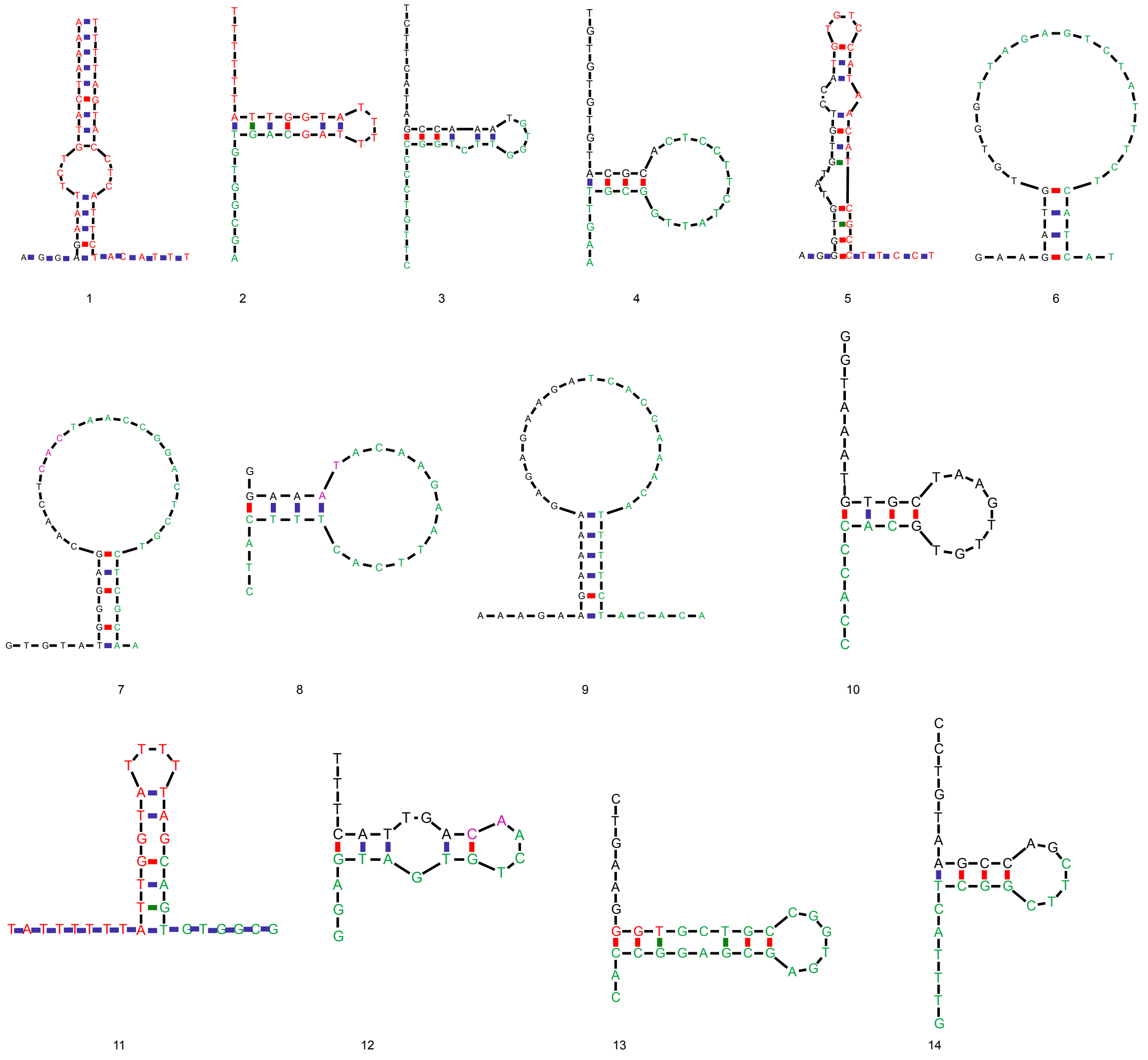

Supplementary Fig.3

D. Small cell lung cancer (SCLC)

1 TTATAGTTGAGTTAATAAAGGGAGCCCTAAATTTGGTTTTGC

2 TGAGGCTCCTAAATCCATCTCCTGTTTTGAGGGAGATTCACCTACAGCT

3 GAAGAGCCTCTAGTTCAGAGCGTCAGTGTTTGTCCCTTTT

4 TCCTCTTTCTATTATGTAGACATAATAGACATGCCTGGGTGTGTCTGTGA

5 CTTGGTGAGCCCGAGACTGGCTGCTTTAGCTATAGGGGCATT

6 GGTGGTAGCAGTTGAATCCAGCCAGCCCCTACCACCAAGCCATGGGCTTGGTACCCCATACCATCCTGTTTCA

7 AACCCTGTGAGATAAATGTGGACAAGAGCTAACTCCATCATGAGATAGCACCGAGGGGATGGTGCTAACCATTCATG

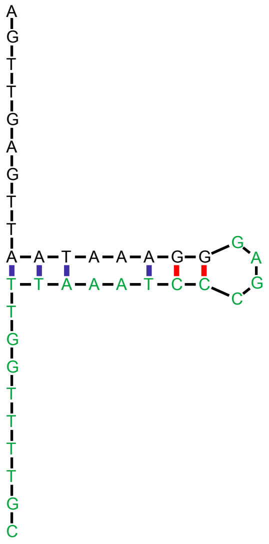

1

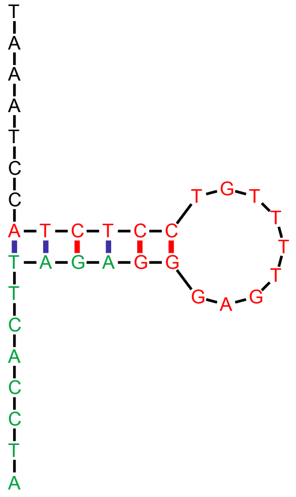

2

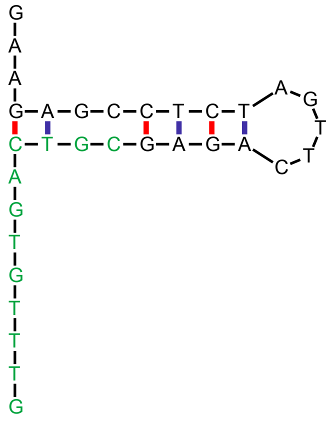

3

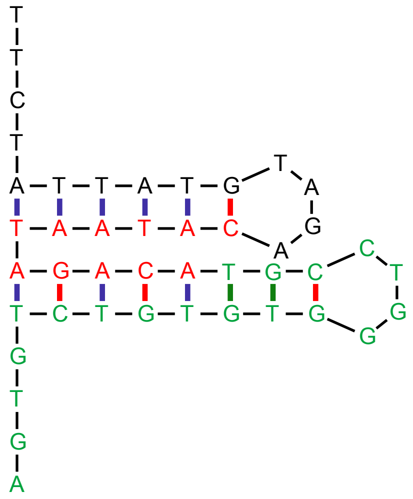

4

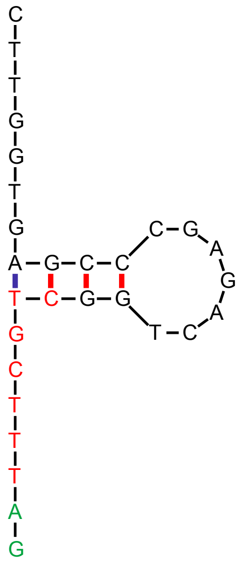

5

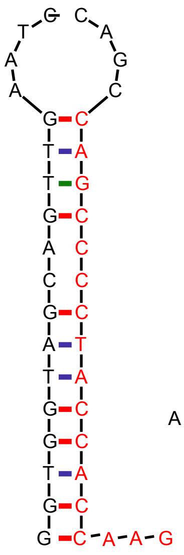

6

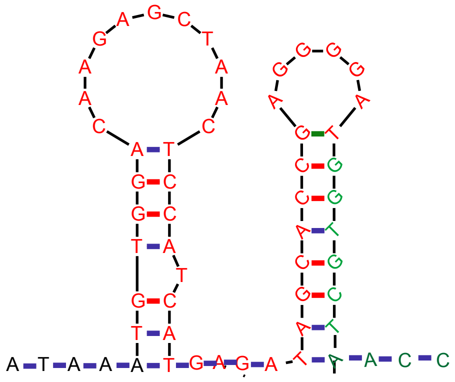

7

Supplementary Fig. 4

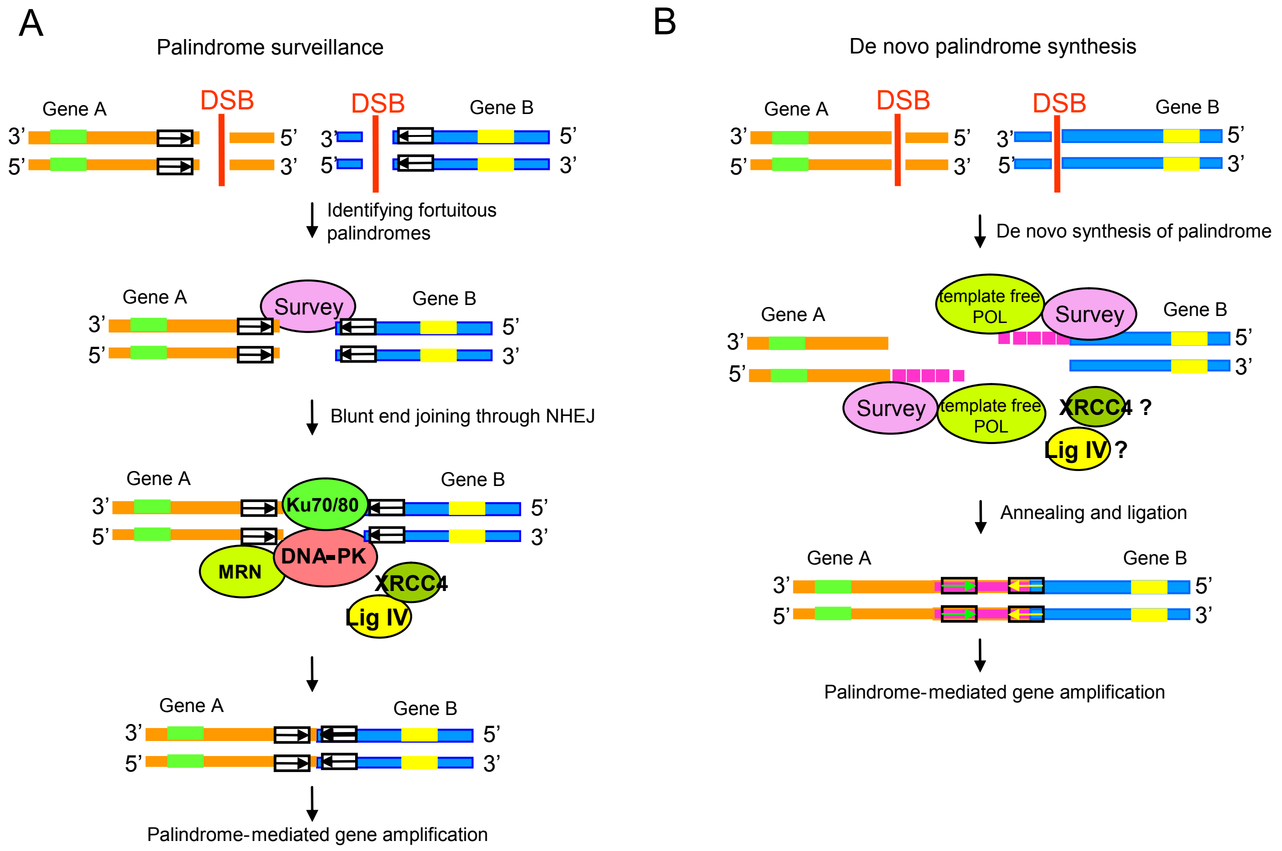

Supplement: Supplementary file 1 [file ijc0133-0797-sd1.pdf]
